# Supplementary material for: Approximate calculations of the net economic impact of global warming mitigation targets under heightened damage estimates
Source: PLoS One. 2020 Oct 7;15(10):e0239520. doi: 10.1371/journal.pone.0239520 (PMC7540901; doi:10.1371/journal.pone.0239520)
Supplement: Attachment — Submitted filename: Response to reviewers.docx [file pone.0239520.s001.docx]

Key

black font = reviewer/editor comment

green font = response to reviewer comment

**Editor**

I have found especially relevant the point raised by the Reviewer#1 about the availability of the code that you have used for your computations. Making the code available is something that we encourage in PLoS ONE and necessary to assure the full reproducibility of published works. Therefore, if you have written sensible parts of code to perform this study, it would be needed that you include them with the manuscript (e.g., linked in a Zenodo repository).

**Response:** We have placed the Matlab scripts that can be used to reproduce the analyses in an online repository, now documented in the acknowledgments.

**Reviewer #1**

In their manuscript "Approximate calculations of the net economic impact of UN global warming mitigation targets under heightened damage estimates" the author compares costs of climate mitigation to the climate damages avoided by those measures. He does so by including an empirically derived damage function into the simple integrated assessment model DICE for a set of linear mitigation curves. With the new damage function (compared to the one used in "classical" DICE) the author finds the costs of climate change (measured in global GDP losses) to outweigh the mitigation costs sooner. Net costs in such a comparison, however, are shown to highly depend on the discount rate as well as the time frame considered.

Overall the manuscript underlines the importance of looking at the time scales of mitigation costs and climate damages when comparing the two. It is written in a clear language and well structured in principal. I have, however, some concerns with the methods and the manuscript.

**Response:** We thank the reviewer for the thorough review and comments. The reviewers’ insight that our main finding has to do with how net costs are highly dependent on the timeframe has inspired us to emphasize this point more in the revised manuscript.

The underlying idea here is to compare climate damages avoided to the necessary mitigation costs. In doing so separately, it is quite similar to the method in Uekerdt et al. [1], who also use Burke et al. 2018 on SSP projections to derive climate damages. They compare these to mitigation costs as per a process-based IAM (REMIND); whereas this manuscript establishes an ensemble of the latter. I very much wonder why [1] get lower optimal temperatures (though when minimizing lost welfare) compared to these results. The author should definitively discuss these differences.

Another recent publication with a similar endeavor the author should relate to - at least in the discussions - is Glanemann et al. [2]. They also incorporate Burke et al. 2018 damages into DICE, while separating growth from level effects in the full optimizing mode of DICE. They find the 2°C target to be optimal, even lower temperature targets depending on the SSP.

This manuscript nevertheless adds to the literature by looking at some uncertainties in the mitigation cost curve, but the aforementioned differences to other parts of the literature should be discussed thoroughly.

**Response:** We thank the reviewer for pointing us toward Uekerdt et al. 2019 and Glanemann et al. 2020. Interpreting our results in the context of these two papers brings out the main theme of our analyses which has to do with the timescale for which the benefits of mitigation are accrued. This is discussed on lines 603-622* in the revised manuscript.

*note that all line numbers refer to the clean revised manuscript not the tracked changes document.

Unfortunately, many important details of the methodology are missing, which makes it difficult to fully follow the conclusions. Ideally, the author would also provide the underlying source code used.

I was very astonished to see that the author does not seem to use DICE in its standard mode of optimization, but rather uses some of its equations. The most important control variable of DICE, actually, is the savings rate, not the mitigation control rate as stated here.

That also the savings rate is derived exogenously, so there is no optimization at all, only became clear to me later throughout the paper. As this is a very important aspect of the methodology here, it should be stated clearly and early.

**Response:** We thank the reviewer for encouraging more clarity on these points. In the revised manuscript we communicate our methodology much more prominently in many places including the abstract and introduction, the methods section and the discussion/conclusion. We have also archived the code used and linked to it in the acknowledgments.

Overall, it is not clear which variables are exogenous and which are endogenous. Also, though some equations can be found in the respective DICE description papers, this manuscript would much benefit from describing its methodology full for the important variables used here.

**Response:** In the revised manuscript we have greatly expanded the methods section. In particular, we have added 3 schematic figures which show how we alter the traditional DICE model in two different ways for our purposes (Figs 1-3 in the revised manuscript). All relevant equations are shown in these schematics and a distinction is made between which variables are exogenous and which are endogenous. All specific parameter values can be found in the archived scripts.

This includes:

- How is the capital depreciation rate used in K(t)? K(t) is determined using the SSPs (eq. 7). Is that only to derive the savings rate? What are typical savings rates derived from the SSPs?

**Response:** In the revised manuscript we clarify that we need to derive the K(t) and the savings rate as a function of time only so that we can calibrate the Burke-DICE damages and that after we have parameter values for our Burke-DICE damage equations, we use the standard DICE value for the saving rate (25.8%). See section 2.4.1 of the revised manuscript.

- How do beta and xi (eq. 8) evolve? Is that from the original DICE? How are they scaled later in the Monte Carlo simulations? Same factor for the whole time series?

- What are the values for the parameters used here, e.g. gamma in eq. 4, c or theta in eq. 8?

**Response:** In the revised manuscript we make clear that we do not alter the mitigation cost equation from its representation in traditional DICE and that our purpose for discussing it is simply that it features prominently in our analyses (it is the cost side of the cost-benefit calculation).

The standard exogenous beta(t) and xi(t) time series are used in the simulations and the values for all parameters can be found in the archived code.

In the monte-Carlo simulations, these time series are altered via a change in their growth rates (see table 1 in the revised manuscript).

- When is Y(t) used and when GWP(t)? Is the former used as gross GWP and the latter as net GWP?

**Response:** Y(t) referred to output prior to its inhibition from damages and mitigation costs while GWP referred to output after it was subjected to damages and mitigation costs. This is clarified in the new Figs 1-3 and we no longer refer to Y(t) in the text.

- For sake of completeness a summarizing equation for the values depicted in the figures (e.g. climate damages, damages avoided) would be very helpful.

**Response:** The new Figs 1-3 should clarify our alterations to the damage module of DICE (and also make clear what is not altered). Additionally, the new equation [2] should clarify how we arrive at our present discounted value of gross world product calculations which inform Figs 10-13.

- Is only SSP2 used throughout the manuscript after Figure 1?
The method description needs to be much more complete before a publication of this manuscript.

**Response:** In the revised manuscript we have clarified that this exercise of using the SSPs was only for the purpose of calibrating the DICE-Burke damage module (Section 2.4.1) and that the remainder of the calculations throughout were conducted under standard DICE2016 gross world product and population assumptions (lines 264-292).

A main point in this manuscript is the ensemble of mitigation scenarios, i.e. time series of mu(t). I wonder how narrowing the assumption of linear mu(t) actually is (assuming a constant reduction rate, i.e. exponential mu(t) seem to be as or even more reasonable). All of these include negative emissions, but for a more complete picture other mu(t) with ambitious mitigation without negative emissions would be highly desirable. Omitting negative emissions in a mu(t) additionally prevents shifting avoided damages into the future and would thus contribute to a more complete picture when looking at the relevant time frames as done in this paper.

**Response:** We agree that these additional experiments would be valuable and that they could be the focus of future work. However, adding an additional set of experiments without negative emissions to this paper would effectively double the results to be analyzed and we feel that this would overburden the reader – especially since the paper is already on the long side.

Especially for communication in Figure 4 the method would benefit from sampling the mu(t) to have equidistant temperatures in 2100 (as that is the main variable used in the subsequent analysis). Regardless of the sampling used, a full description of the parameters used for that must be given in the method description.

**Response:** Due to the specifics of our methodology, we are unable to prescribe the temperature in 2100 and thus we are unable to force the sampling to be equidistant in temperature in 2100. However, the resolution of temperature in 2100 under our methodology is sufficiently high (within 0.1C, Fig 8b) to be able to discuss results as a function of temperature in 2100 (Fig 9 and elsewhere).

In lines 150-158 the authors states three primary advantages of the method used. I partially agree with the first two, though I think one should note that, since DICE is not used in its optimizing mode, the results of this paper are not easily transferable to "normal" DICE analyses.

**Response:** In the revised manuscript we add this point on lines 471-473.

However, the author claims that the method "removes the philosophical complications associated with converting consumption to human well-being via a utility function" (3rd point). While this is always a problematic issue, it is not avoided here at all. Rather than focusing on well-being via a utility function, this study looks at net production (Y/GWP), which is implicitly assumed to be the relevant measure to be maximized for this cost-benefit analysis (or "to justify the [...] temperature targets" as stated in the abstract). In a revised article this needs to be made more explicit.

**Response:** This is an important point and we thank the reviewer for highlighting it. In the revised manuscript we discuss this issue much more extensively on lines 94-210.

Throughout the revised manuscript, we change the language so that we are not overclaiming about the “justification” of temperature targets and rather we emphasize that we are simply doing calculations on gross world product.

Additionally, maximizing Y/GWP in the optimization mode in DICE would essentially mean no consumption. One might thus ask, if sticking to gross production as per the SSP data with a fixed savings rate makes sense when, at the same time, comparing mitigation costs and avoided damages in terms of GWP; in most IAMs this would result in shifting consumption in time additionally avoiding climate damaging production.

**Response:** We agree that maximizing gross world product at the expense of consumption would be a nonsensical outcome of optimizing around gross world product. In the revised manuscript we articulate this as one of the reasons for running our DICE model with exogenous control rates trajectories (lines 167-169). Overall, the methods should me much clearer in the revised manuscript.

On a minor note, I would very much appreciate a short discussion on the concept of the discount rate. Since the costs of climate change go sub-linear in time (Figure 4d), a sufficiently large discount rate (as an exponent for exponentially diminishing sum) will always outweigh them (Figure 7); regardless of the time horizon.

**Response:** In the revised manuscript we have added a discussion of the concept of discount rate on lines 447-458 and we discuss the implications of a sufficiently large discount rate outweighing the net benefits of mitigation on lines 523-535.

Overall, I believe, when already using an integrated assessment model, the question of cost-benefit would be much better tackled in the full optimizing mode. This would actually embrace the idea of normative assessment in inter-temporal optimization for which such a model is built. Deriving both cost curves independently as done here, totally neglects their interaction (perceived damages in the future). Nevertheless, after a thorough revision, this publication could definitely contribute to the literature on climate change cost-benefit analyses.

**Response:** In the revised manuscript we have expounded upon our reasons for running the model under a spectrum of exogenous control rates. Specifically, it has to do with the primary impetus of the study being an investigation of the ~36 trillion US$ avoided damages figure from Burke et al. (2018) which is for a finite time horizon of 2100 (see lines 94-210).

Additionally we refer readers to the closely-related paper referenced by the reviewer (Glanemann et al., 2020) that does indeed run DICE in optimization mode after adding Burke et al. 2015 – like damages.

Other comments/questions:

Abstract (lines 13/14)

- I doubt that's a reasonable interpretation of the "break-even year" as used in this manuscript. Here it is the year in which the mitigation costs of that year and those of the damages avoided (for that year!) are the same. This notion totally neglects that the avoided damages of a certain year - due to the inertia in the climate system - result from mitigation efforts before that year. Thus, this definition of "break-even year" is only useful in a technical sense.

**Response:** In the revised manuscript, we have removed this particular interpretation of the break-even year from the abstract.

Figure 1:
- Please use a legend instead of arrows
- Do you mean "GWP change" (as it is <0, which is meant to be losses)?

**Response:** Yes, we have changed this to gross world product change. We have considered using legends in all cases where the reviewer requests them but in some cases we believe arrows to be necessary and have retained them.

Figure 2:
- Are the values on both y-axes "per year" or "per 5 years"?

**Response:** We have added this information in the figure caption.

Figure 3:
- Which discount rate is used here?
- Please use a legend instead the arrow
- "loss" in x-axis label should be removed

**Response:** We have added the discount rate to the figure caption. We have retained the word loss as we think it is clarifying.

Figure 4:
- Please use a legend instead of arrows
- Damages avoided would be an interesting variable
- Please mark y=0 in c & d. Where do damages start?

**Response:** We have added y=0 in c and d showing that damages start below 1%.

Line 126: Capital C?

**Response:** This equation is now in the schematic Figs 1-3 and it has been corrected.

Line 248: What about renewable energies?

**Response:** We have add “like renewable wind and solar energy” to this sentence.

Line 558: "[...] it is controversial as to whether historical temperature variability is an appropriate analogue for the economic amages to be expected from sustained climate change": The same argument could be used to argue that the climate damages are underestimated

**Response:** In the revised manuscript we have a discussed how both of the damage estimates considered in our paper may be over or underestimated (lines 77-84, 258-262, 633-654).

Line 568 "[...] or over time": Looking at the distribution of the benefits and costs over time is the point of this manuscript, is it not?

**Response:** Thank you, yes this was a typo.

References:

[1] Ueckerdt et al. The economically optimal warming limit of the planet. Earth Syst. Dyn., 2019
[2] Glanemann et al. Paris Climate Agreement passes the cost-benefit test. Nat Comm., 2020.

**Reviewer #2**

I would like to thank the author for a very interesting article on a very topical matter. The paper is extraordinarily well written and very easy to follow. Given the large literature on DICE modelling, I think the paper adds a number of interesting aspects, such as comparing DICE outcomes to the SSPs, yet before this paper is suitable to publish, I would, recommend a number of changes, clarifications and additional discussions to be taken up by the author.

**Response:** We thank the reviewer for the encouragement and the thorough review.

Substantive Comments

1. It seems to me that the author is making a number of highly interesting additions to the DICE modelling framework. Yet overall, the fundamental question the author intends to answer (are the Paris Agreement goals economically beneficial, given new information on the damage relationship?) does not necessitate all these modifications to the DICE set-up. As DICE, in its original form, already equates costs and benefits and hence answers this question, I would be highly interested in the result of DICE with an amended damage relationship, but keeping the emission control rate endogenous, rather than as an exogenous variable derived from the RCPs (see Glanemann et al. 2020, Nature Comms).

**Response:** In the revised manuscript we have expounded upon our reasons for running the model under a spectrum of exogenous control rates. Specifically, it has to do with the primary impetus of the study being an investigation of the ~36 trillion US$ avoided damages figure from Burke et al. (2018) which is for a finite time horizon of 2100 (see lines 94-210*).

Additionally, we refer readers to the closely-related paper referenced by the reviewer (Glanemann et al., 2020) that does indeed run DICE in optimization mode after adding Burke et al. 2018 – like damages.

*note that all line numbers refer to the clean revised manuscript not the tracked changes document.

2. In line 413, the author contrasts his results to Burke et al (2018), who find achieving 1.5°C over 2°C would result in $36tn economic benefits by 2100, while archiving this target would result in a loss of $9tn in GWP under the author’s framework, which also includes mitigation costs. This would imply mitigation costs of $45tn by 2100 – I would urge the author to discuss whether such a value for mitigation costs seems plausible.

**Response:** In the revised manuscript we emphasize that the mitigation costs in DICE are calibrated to those of the most recent processed-based IAMs and thus they should represent a mainstream estimate. Nevertheless, we add a brief discussion of this $45tn mitigation cost number in the context of a recently-released study:

van Vuuren, D.P., van der Wijst, K., Marsman, S. et al. The costs of achieving climate targets and the sources of uncertainty. Nat. Clim. Chang. 10, 329–334 (2020). <https://doi.org/10.1038/s41558-020-0732-1>

This study gives a range of $10tn to $100tn of mitigation costs associated with 1.5°C by 2100 at a 5%/year discount rate (relative to no mitigation). $45tn would be on the high end of their estimates when comparing 1.5°C to 2.0°C but within their large uncertainty range (lines 481-484).

3. The author stresses that the goal of his study is to represent the range of uncertainty between both DICE versions (Line 115). I would suggest representing this intention in the abstract as well, as uncertainty is not currently addressed in the abstract.

**Response:** We tried versions of the abstract that made this point but ultimately decided that they distracted from our primary points. Nevertheless we make sure to emphasize this point prominently elsewhere in the manuscript (e.g., lines 77-84, 258-262, 633-654).

4. The author transparently states that in his framework μ(t) must take a linear form. I would appreciate it, if the author would discuss the validity of this assumption based on empirical data (most process-based IAMs tend to suggest an inverse functional relationship being needed/most realistic) and some discussion whether his results would hold under an amended functional form.

**Response:** In the revised manuscript we have added a figure that shows the linear μ(t) trajectories in the context of μ(t) curves that have been calculated under optimization mode (Fig 4). This shows that the typical optimized pathway is not very far from linear. Also on lines 655-661 we make the point that despite not being completely optimal, this framework is likely to produce more economical emissions reductions pathways than real world scenarios that would involve more policy inefficiencies.

5. It remains unclear to me whether the values of a, b, and c were tested for their sensitivity. It seems to me they were not included in the Monte Carlo analysis. As they are crucial values for the subsequent analysis, I would urge the author to discuss whether the choice of values (both individually as well as in relation to each other) would affect the estimation result. Further, I would appreciate a comparison of the study’s values with the empirical evidence of temperature effects on capital depreciation as well as on total factor productivity.

**Response:** In the revised manuscript we expand greatly the calibration of the Burke-DICE version of the model and on the details of how a, b, and c were calculated (lines 264-292). We also emphasize that the values were chosen so that damages on gross world product would match those produced in Burke et al. (2018). Finally, we note that these functional forms were informed from empirical fits on economic growth and not from established theory on how temperature affects capital depreciation (lines 245-249). Along these lines, we site a study on the relationship between total factor productivity and temperature (Letta et al. 2018; our new ref. 64) that suggest the Burke et al. (2018) growth effects may be overestimated (line 259 and line 652)

Minor Comments
6. The author should detail the exact data source for Figure 3. I am unclear how the four boxes of for the mitigation costs of RCPs have been calculated and which IAM variable in process-based variables is used for this comparison (I assume the data was taken from the Appendix of reference 65, but more detail would be appreciated).

**Response:** In the revised manuscript we have expanded on the analyses that underlies this figure and we have documented the exact source of the data is from Fig S2 in our ref. 46. (lines 345-347).

7. I recognise that the author is following Moore and Diaz in their functional form for equations 5 and 6, but I would appreciate an explanation/discussion on why temperature enters linearly in 5 and in a non-linear form in 6.

**Response:** In the revised manuscript we state that these functional forms come out of the empirical analysis of Dell et al. (2012) which is discussed in the supplementary material of Moor and Diaz (2016). That is, the linear reduction in A(t) with temperature and the nonlinear increase in capital depreciation rate with temperature are not necessarily based on theory but rather a calibration of these variables to the empirically-derived relationship between temperature and growth (lines 245-249 in the revised manuscript).

We also note that the specific partitioning of impacts between A(t) and δ(t) is not particularly important for our purposes. Rather, we are primarily concerned with implementing a parameterization that results in damages that are consistent with the associated globally-aggregated damages calculated by Burke et al. 2018 (lines 255-257).

8. The goals the article is referring to are not “United Nations” goals. They are specific to certain international treaties and declarations, which have varying membership but are not universally accepted by all member states of the United Nations. In the spirit the author is referring to them, they should be referred to as “Paris Agreement” targets. Similarly, the Paris Agreement did not “affirm” the 2°C target (line 55), as it was enshrined in an internationally legally binding document for the first time then (Copenhagen Accord was not a treaty), while the language was strengthened in the PA to “well-below 2°C” rather than “below 2°C”.

**Response:** We appreciate this clarification and have changed the language throughout the manuscript (as well as the title) to be in line with these recommendations.

9. For readers unfamiliar with the economics terminology, I would suggest explaining all variables used in equations when they first appear – even if they are as simple as labour, capital and Utility (line 126, the definition is only provided two pages later).

**Response:** In the revised manuscript we have greatly expanded the methods section. In particular, we have added three schematic figures (Figs 1-3) which show all relevant equations and define all relevant terms.

10. In Figure 1, the author could consider presenting SSPs 1,3,4 and 5 in an appendix.
Where possible, Table 1 should feature the Greek letter for the variables in the Monte Carlo analysis as well.

**Response:** We considered placing some results in an appendix but ultimately we decided that the ideas are better communicated in a single figure. We have added the Greek letters to Table 1.

11. In line 296, rather than referring to “comprehensive” IAMs, I would stick to the term used in line 258 “process-based” (alternatively: energy-system models or cost-effectiveness IAMs).
Reference 47 has different formatting to all other references.

**Response:** We have changed the word “comprehensive” to “process-based” throughout the revised manuscript and updated the reference formatting.

12. The author could consider citing Lontzek, TS; Cai, Y; Judd, KL; et al. 2015 when discussing possible non-linear changes in the climate system and their effects on DICE modelling.

**Response:** In the revised manuscript we cite this paper on line 82.
