# Supplementary material for: Approximate calculations of the net economic impact of global warming mitigation targets under heightened damage estimates
Source: PLoS One. 2020 Oct 7;15(10):e0239520. doi: 10.1371/journal.pone.0239520 (PMC7540901; doi:10.1371/journal.pone.0239520)
Supplement: Attachment — Submitted filename: Response.docx [file pone.0239520.s002.docx]

Patrick T. Brown, PhD

Assistant Professor, Department of Meteorology and Climate Science,

San Jose State University, San Jose, CA

Response to Reviewer Comments RE:

Dear Dr. Añel,

We would like to thank you and the reviewers for evaluating and providing feedback on our revision. We believe that this additional iteration of the review process has further improved the manuscript.

A tracked changes document is included and a point-by-point response is below.

Key

black font = reviewer/editor comment

green font = response to reviewer comment

**Editor**

I would ask you to check the comments by a new reviewer carefully. Some of them can seem awkward, however, they go to the point, and in my view, there are several issues raised need a good rebuttal.

**Response:** The most substantive comment raised by the new reviewer is that we have not removed the influence of normative parameters like the risk aversion parameter (the elasticity of marginal utility, η) in our configuration. This is incorrect. As Figures 1-3 show, we do not have any version of a risk aversion parameter in our configuration. The reviewer is incorrect to insinuate that our results still implicitly contain this parameter and that we have simply set its value to zero. We elaborate on this in our response to the reviewer and in the revised manuscript.

You could consider necessary running further experiments to check the impact of changing parameters.

**Response:** Since there is no risk aversion parameter in our configuration, our results cannot be sensitive to its value.

The sensitivity of our results to the other major normative parameter, the discount rate, is tested and discussed extensively, especially in the text pertaining to Figure 11.

**Reviewer #1**

N/A

N/A

**Reviewer #2**

I would like to thank the authors for implementing many of the suggested revisions from my first set of comments. Especially welcome is the revamped methodology section and especially the included schematic figures ensure clarity much better.

**Response:** We thank the reviewer for encouraging these changes and improving the manuscript.

Given these changes, I only have a few remaining comments, which are either editorial or mainly concern the paper’s framing. The framing of the paper is ultimately of course up to the authors, but I would encourage the authors to carefully consider my major comments, as I think the possible reception of this paper from certain audiences might deviate from the paper’s actual conclusions.

This overall leads me to conclude that I think this is a highly interesting and well-presented paper which ultimately should be published, but I do believe that the authors should be wary of the reception of their results, which is why I suggest a few small changes to ensure adequate qualifiers are added, where they are necessary.

Major comments:

I understand that the authors have chosen to report a particular specification of discount rate and time preference as their main result in order to compare their results to Burke et al’s number of US$36tn. However, considering the major implications their claims could have regarding the 1.5°C and 2°C targets independent of Burke et al’s result, I would urge the authors to qualify their 2100 main result with reference to the considerable sensitivity that is highlighted in their paper. The authors themselves argue that the sign of the 2100 result is sensitive to variation in their parameters and that across reasonable parameters the negative result would not hold for e.g. 90 or 95 per cent of the analyzed parameters (the authors list that this is the case for 70% of the 2°C result and 80% of the runs for the 1.5°C result in their Burke-DICE) – in contrast to the 2300 result where the sign of the estimated effects seems to be clear. I would suggest to the authors to qualify their main result for 2100 in the appropriate places (e.g. abstract, conclusion, etc.) with a reference to a certain sensitivity to the parameters chosen.

**Response:** In the revised manuscript we have added language to the abstract that indicates that our main conclusion only holds under most parameter combinations considered (line 17). Our discussion and conclusions section discusses these and many other important caveats.

In a similar vein, I would argue the authors should at least mention that their calculations are not cost optimal pathways in the abstract and the conclusions.

**Response:** In the revised manuscript we express on line 14 in the abstract and line 672 in the conclusion that the experiments are run under exogenously-defined mitigation stringency pathways and not in optimization mode.

I thank the authors for adding the reference that now clarifies that US$ 45tn is a reasonable estimate for the mitigation costs; at least according to the existing literature. Perhaps the authors could add whether this is also the number that their model uses for the mitigation costs based on the SSPs. Currently, the authors simply compare their overall GWP loss to Burke et al’s damage statement, which implies the US$45tn mitigation cost. In effect, I would be interested to see how close the authors parameterization of the damages gets to Burke et al’s original number.

**Response:** In the revised manuscript, we have added 3 paragraphs of discussion on this (lines 482-505).

Minor comments:

I would appreciate if the authors could restate in one sentence or so how Burke et al 2018 have arrived at their US$36tn figure so as to make the comparison easier (around line 480).

**Response:** In the revised manuscript, we are clearer on the details of the Burk et al. 2018 calculation (line 478).

It is not entirely clear which study the authors are basing their damage parameters from. In a number of places, they refer to Burke et al 2015 and in others to Burke et al 2018. I would guess that their reference to Burke et al 2015 is wrong e.g. in Fig 3 caption, line 237, etc.

**Response:** In the revised manuscript we have changed the references in the Fig. 3 caption and line 237 to Burke at al., 2018.

In the legend for Figure 1, the authors forgot the parameter h

**Response:** There are a few variables that are not listed in the key which are explained by the new caption sentence: “Variables not defined in the key are constants. See [47] for further details.”

I thank the authors for making their replication code available. Before publication, however, they should ensure to add replication instructions to the GitHub repository.

**Response:** We have eliminated unnecessary scrips and added replication instructions in the comments of the main figure-producing script.

Reference 87 seems to have a formatting issue regarding the names of the authors in the bibliography.

**Response:** Thank you we have fixed this.

**Reviewer #3**

I did not review the previous version of the paper. The authors worked hard to revise the paper, but more work is needed. The language is awkward, the authors mess up their welfare theory, and the rely on a wrong model (see below).

**Response:** We have revised the awkward language that the reviewer pointed out.

However, the reviewer is incorrect that we mess up our welfare theory. In fact, an advantage of our approach is that we have no welfare theory and that our results are not sensitive to the risk aversion parameter that goes into a welfare function. We also extensively test the sensitivity of results to the discount rate (Figs. 10, 11, 12 and 13).

Regarding Burke et al. 2015/2018, being a “wrong model”, we argue that it is one of the most influential damage estimates that exists and definitely worth interrogating.

We elaborate on both of the these points below.

Furthermore, there is little new in here. The authors refer to a series of papers that do very similar things.

**Response:** What is new is incorporating the Burke et al. 2015/2018 damage function into DICE and directly comparing the gross world product claims of Burke et al. 2018 against a mainstream mitigation cost estimate. This has not been done before to our knowledge.

Additionally, we note that the editorial policy of PLOS ONE states: “PLOS ONE substantially cuts time to publication by eliminating subjective assessments of significance or scope to focus on technical, ethical and scientific rigor”. We find the reviewer comment to be a subjective assessment of significance or scope. We would appreciate the opportunity to have the significance of our work judged by the wider research community.

"Traditional DICE" Traditional? Please consult a dictionary.

**Response:** According to dictionary.com the word “traditional” refers to “existing in or as part of a tradition; long-established”. This is exactly what we mean to communicate by this word. There is a very rich literature using the DICE model in the configuration shown in Fig. 1 and thus this configuration qualifies as “long-established”. Furthermore, there should be no ambiguity as to what we mean when we use the word traditional since there is an entire section dedicated to explaining precisely what we mean (Section 2.1).

"we find it simplifying" ugly language

**Response:** We have removed this phrase.

"to eliminate the social welfare function [...] so that the specific values of the normative parameters are not latently affecting our GWP calculations" what is "latently affecting"? more importantly, you did not remove the normative parameters, you set the rate of risk aversion to zero. You may want to reread Bernoulli's work on this.

"It eliminates the latent influence of normative parameters" No, it does not. It just replaces one set of normative parameters with another.

**Response:** We have removed the word “latently” for clarity.

We have indeed removed the normative risk aversion parameter (η) from influencing our results. This is signaled by the parameter’s absence in Fig 2 and Fig 3. Specifically, as we state on line 196 that “we are using equations in DICE to run forward-projections in the same way a typical climate model would be run rather than using the equations to inform the perspective of a benevolent social planner.” Thus, the η parameter cannot influence the results because the model is not doing any calculations that depends on it.

We could reintroducing the η parameter by converting gross world product to consumption and consumption to utility and utility to total social welfare. However, this would be a confusing distraction since the variable that is the main topic of the paper is gross world product and our methodology intentionally avoids having η influence our calculation of gross world product.

Our results are of course influenced by the discount rate but the sensitivity of the results to the discount rate is already discussed extensively around figures 10, 11, 12 and 13.

The proposed method is not at all new. In the literature, FUND and PAGE have been running in this mode for decades.

**Response:** We appreciate that the reviewer notes that there is precedent for running IAMs in a similar configuration as we do. We do not claim that our configuration is some sort of innovation (line 184), we simply claim that it confers advantages (Section 2.2).

"pretext" see above remark about a dictionary

**Response:** This was indeed an error and we have changed the word to “preamble”.

"suggest" is the right word -- there is little evidence that climate change affects the growth rate of output rather than its level -- I do not need to point you to the relevant papers, which are in your bibliography.

The Burke model is not credible. It is by now common knowledge that there econometrics are wrong. They claim to regress a stationary variable (the change in log per capita income) on a non-stationary one (temperature). In fact, the regress income growth on the cointegrating vector of temperature and year dummies. This works in-sample, although their parameters are biased and their standard errors meaningless. Out-of-sample, the model goes quickly off the rails.

**Response:** We make it clear that the purpose of our paper is not to assess how realistic the Burke et al. 2015/2018 damage estimates are (lines 259-263 and 665-671).

We disagree that it is “common knowledge” that the Burke et al. 2015/2018 model is not credible. Burke et al. (2015) has been cited nearly 1,000 times and it has been cited [140 times in the 1^st^ half of 2020 already](https://scholar.google.com/scholar?as_ylo=2020&hl=en&as_sdt=0,24&sciodt=0,24&cites=7238780295604215172&scipsc=). The reviewer makes it seem like our work is not worth doing because nobody takes the Burke et al. 2015/2018 damage function seriously. We find this to be demonstrably false.

We encourage the reviewer to publish their methodological critiques of Burke et al. 2015/2018 so that they can be more widely appreciated.
