# Supplementary material for: Approximate calculations of the net economic impact of global warming mitigation targets under heightened damage estimates
Source: PLoS One. 2020 Oct 7;15(10):e0239520. doi: 10.1371/journal.pone.0239520 (PMC7540901; doi:10.1371/journal.pone.0239520)
Supplement: Attachment — Submitted filename: Brown_Saunders_Temp_targets_PLOS_Response_3.docx [file pone.0239520.s003.docx]

Patrick T. Brown, PhD

Assistant Professor, Department of Meteorology and Climate Science,

San José State University, San José, CA

Response to Reviewer Comments:

Dear Dr. Añel,

We would like to thank you and the reviewer for your further comments. We believe that this additional iteration of the review process has further improved the manuscript.

A tracked changes document is included and a point-by-point response is below.

Key

black font = reviewer/editor comment

green font = response to reviewer/editor comment

**Editor**

I think that the comment by the reviewer about toning down the statements included in the paper is right, and you should adopt it.

**Response:** We agree. In the revised manuscript, we added language to the abstract (line 10), introduction (lines 104-106) and conclusion (lines 701-702) that indicates that our analysis builds heavily off of previously-published work. We make no claims that our methodology is pioneering or particularly novel. The lines added to the introduction are reproduced below:

*Our study follows in the footsteps of several previous studies that incorporated damages to economic growth into DICE [21, 32, 33, 44] but we seek to address the following specific questions.*

Also, I would encourage you to at least discuss better the limitations of the Burke model in the text and their potential impact on your results. Maybe including a brief specific subsection on this would be a possibility.

**Response:** In the revised manuscript we have added a subsection (2.4.1) on the limitations of the Burke model (lines 263-279) which is reproduced below:

***2.4.1 Validity of the Burke et al. 2018 damage estimates***

*It should be noted that the severity and validity of the damage estimates from [39, 43] has been disputed [40, 65, 66]. Specifically, [66] use a different statistical model specification in their historical temperature - gross domestic product regressions and project a much smaller amount of damages per degree of global warming by 2100 than [39, 43] (approximately a 7% loss under a no-mitigation scenarios as opposed to 23%). [65] also use a different statistical model specification than [39, 43] and show a relatively small influence of temperature of economic growth (Total Factor Productivity growth in particular) and only in low-income countries. Finally, [40] show that the best performing statistical models in an out-of-sample test relate temperature to gross domestic product levels rather than gross domestic product growth. They conclude that the specifications of [39, 43] are almost certainly not the optimal specifications and they argue that more accurate specifications amount to economics damages by 2100 of only 1-2%.*

*Despite these countervailing results, even if [39, 43] overestimate the impact of temperature deviations on gross domestic product historically, it would still be possible for GWP damages to reach the aggregate levels projected by their model by 2100 if the damages come about through pathways other than directly from temperature (e.g., a large sea level rise due to the collapse of the West Antarctic ice sheet).*

The last point above is important because it means that even if it were definitively proved beyond all doubt that the Burke model overestimates the relationship between temperature and GDP growth, it could still be the case that damages reach the amount projected by Burke by 2100 and thus the calculations in our paper would still be valuable.

**Reviewer #1**

N/A

N/A

**Reviewer #2**

N/A

N/A

**Reviewer #3**

Dismissing comments will get you nowhere.

**Response:** We are working hard to address all the concerns from reviewers.

Moyer et al., Moore and Diaz, Dietz and Stern and Ricke et al. have all done very similar things. You need to tone down your claim of novelty.

**Response:** In the revised manuscript, we added language to the abstract (line 10), introduction (lines 104-106) and conclusion (lines 701-702) that indicates that our analysis builds heavily off of previously-published work. We make no claims that our methodology is pioneering or particularly novel.

The Burke model is wrong. It may be frequently cited, but that does not make it right. You will need to defend your specification. Hiding behind authority, or in this case faux-authority is not an option.

**Response:** The title of the paper is “Approximate calculations of the net economic impact of global warming mitigation targets *under heightened damage estimates*”. We do not claim that the Burke model is correct anywhere in the paper and we take pains to communicate that we are *not* championing its validity (e.g., lines 683-686). Instead our intent is simply to show what the cost-benefit calculations would be *if* the Burke model was correct (i.e. “under heighted damages”). We believe that this is a valuable exercise precisely because the Burke model is so frequently cited and influential.

Nevertheless, we have added a subsection (2.4.1) on the limitations of the Burke model (lines 263-279) which is reproduced above in our response to the editor.
