# Supplementary material for: Approximate calculations of the net economic impact of global warming mitigation targets under heightened damage estimates
Source: PLoS One. 2020 Oct 7;15(10):e0239520. doi: 10.1371/journal.pone.0239520 (PMC7540901; doi:10.1371/journal.pone.0239520)
Supplement: Attachment — Submitted filename: Brown_Saunders_Temp_targets_PLOS_Response_4.docx [file pone.0239520.s004.docx]

Patrick T. Brown, PhD

Assistant Professor, Department of Meteorology and Climate Science,

San José State University, San José, CA

Dear Dr. Añel,

A tracked changes document is included and a point-by-point response is below.

Key

black font = reviewer/editor comment

green font = response to reviewer/editor comment

**Editor**

The reviewer#3 has provided additional comments on your manuscript. I acknowledge that they are exigent. However, I feel that we are moving quickly in the review process and that your paper is being hugely improved. Therefore, I would like to ask you to make an extra effort to address their concerns. You can see their comments below.

**Response:** We agree that the manuscript has been vastly improved by the review process.

We have followed the most recent substantive request from reviewer #3 and added the suggested additional analysis.

It is worth pointing out that if the Burke damage estimates are too high, it only bolsters one of the central points of our manuscript rather than detracting from it. This point is that *even if* damages are as large as what the Burke model suggests, it *still* comes out as a net loss to GWP (this century) to limit warming to the Paris Accord goals. This conclusion is *only strengthened* if the Burke model overestimates damages.

**Reviewer #3**

You now admit that Burke might be wrong, but continue to refuse to spell out what's wrong and what's right with that paper.

**Response:** “You now admit” is a mischaracterization. Our paper never contended that the Burke model was “right”. Our paper contained the following paragraph from the initial submission which communicates that our goal is not to adjudicate which damage estimates are “right” and which are “wrong”:

*“It should be noted that the accuracy of both Burke et al. (2018) and the Default-DICE representations of damages have been disputed [40, 42, 43]. The goal of this work, however, is not to assess whether the Burke-DICE or Default-DICE representation of damages better represent reality. Instead, we interpret the large range spanned between Default-DICE and Burke-DICE damages as a rough proxy of the substantial uncertainty in true economic damages from climate change.”*

The reviewer makes it clear that *they* find the Burke estimates to not be credible and that is their right. But that is not necessarily the widely-held view. Our paper is aimed at those researchers who are not so convinced that the Burke model is wrong. As we stated in a previous response, many researchers take the Burke damage estimates quite seriously – Burke et al. (2015) has now been cited 167 times in the calendar year 2020, and 942 times overall.

But now that you've admitted the mistake, the paper has a serious flaw: You compare Nordhaus-DICE to Burke-DICE. The comparison masks two changes at once: You switch from damages-in-level to damages-in-growth and from credible-damage-estimates to disputed-damage-estimates. (Disputed is a euphemism for not-credible.) You therefore need to add an intermediate case, with damages-in-growth as estimated by Fankhauser, Dell, Letta or Kahn.

**Response:** Many authors dispute the credibility of the Nordhaus damages as well so it is not fair to say that we comparing “credible” to “non-credible” damage estimates. Lines 78-95 in the paper spell out many of the published criticisms of the Nordhaus damage estimates.

We strongly disagree that the paper “masks two changes at once”. We go to great lengths in terms of both text and diagrams (Figures 1-3) to explain precisely what we are comparing and why. Nothing is hidden or masked.

Nevertheless, we have conducted the additional analysis that the reviewer suggests. Specifically, we add a discussion of “an intermediate case, with damages-in-growth” based on the Kahn reference. This discussion appears on lines 480-498 of the revised manuscript and is reproduced below.

*“Additionally, as discussed in Section 2.4.1, the magnitude of the [39, 44] damages to economic growth have been challenged, with other groups obtaining results that do imply damages to growth but damages that are less severe and result in less aggregate impact by 2100. One example is [66] who find damages to growth that would result in a 7% loss of per-capita GWP by 2100 in a no-mitigation case and a 1% loss of per-capita GWP by 2100 under mitigation that results in limiting global warming to 2°C.*

*In order to investigate the net costs associated with damages such as these, we re-tuned the DICE-Burke model (Figure 3) such that the accumulated damages on growth matched those found in [66]. Specifically, we re-tuned the coefficients of the model such that in the no-mitigation case, damages on per-capita GWP in 2100 matched the 7% projected by [66] and in the 2°C case, damages on per-capita GWP in 2100 matched the 1% projected by [66]. (‘a’ was reduced to 1% of its Burke-DICE value, ‘b’ was reduced to 50% of its Burke-DICE, ‘c’ was reduced to 10% of its Burke-DICE and an exponent of 2 was added to the temperature term in the damages to total factor productivity).*

*Using the above damage estimates, we calculate that achieving the 1.5°C and 2.0°C targets result in net GWP losses of ~160 trillion US$ and ~60 trillion US$ respectively, relative to the no-mitigation case through 2100 (which is in between those of Default-DICE and Burke-DICE, Figure 10). This implies a ~100 trillion US$ loss from limiting global warming to 1.5°C relative to 2.0°C which is also in between the ~40 trillion US$ loss calculated under Burke-DICE and the ~120 trillion US$ loss under Default-DICE (Figure 10).”*

These results show that with intermediate damage estimates our results also predictably fall intermediately between Default-DICE and Burke-DICE.

It is worth pointing out that if the Burke damage estimates are too high, it only bolsters one of the central points of our manuscript rather than detracting from it. This point is that *even if* damages are as large as what the Burke model suggests, it *still* comes out as a net loss to GWP (this century) to limit warming to the Paris Accord goals. This conclusion is *only strengthened* if the Burke model overestimates damages.
